# What matters to women during childbirth: A systematic qualitative review

**DOI:** 10.1371/journal.pone.0194906

**Published:** 2018-04-17

**Authors:** Soo Downe, Kenneth Finlayson, Olufemi Oladapo, Mercedes Bonet, A. Metin Gülmezoglu

**Affiliations:** 1 Research in Childbirth and Health (ReaCH) group, University of Central Lancashire, Preston, United Kingdom; 2 UNDP/UNFPA/UNICEF/WHO/World Bank Special Programme of Research, Development and Research Training in Human Reproduction (HRP), Department of Reproductive Health and Research, World Health Organization, Geneva, Switzerland; Universiti Sains Malaysia, MALAYSIA

## Abstract

**Introduction:**

Design and provision of good quality maternity care should incorporate what matters to childbearing women. This qualitative systematic review was undertaken to inform WHO intrapartum guidelines.

**Methods:**

Using a pre-determined search strategy, we searched Medline, CINAHL, PsycINFO, AMED, EMBASE, LILACS, AJOL, and reference lists of eligible studies published 1996-August 2016 (updated to January 2018), reporting qualitative data on womens’ childbirth beliefs, expectations, and values. Studies including specific interventions or health conditions were excluded. PRISMA guidelines were followed.

**Data collection and analysis:**

Authors’ findings were extracted, logged on a study-specific data form, and synthesised using meta-ethnographic techniques. Confidence in the quality, coherence, relevance and adequacy of data underpinning the resulting themes was assessed using GRADE-CERQual. A line of argument synthesis was developed.

**Results:**

35 studies (19 countries) were included in the primary search, and 2 in the update. Confidence in most results was moderate to high. What mattered to most women was a positive experience that fulfilled or exceeded their prior personal and socio-cultural beliefs and expectations. This included giving birth to a healthy baby in a clinically and psychologically safe environment with practical and emotional support from birth companions, and competent, reassuring, kind clinical staff. Most wanted a physiological labour and birth, while acknowledging that birth can be unpredictable and frightening, and that they may need to ‘go with the flow’. If intervention was needed or wanted, women wanted to retain a sense of personal achievement and control through active decision-making. These values and expectations were mediated through womens’ embodied (physical and psychosocial) experience of pregnancy and birth; local familial and sociocultural norms; and encounters with local maternity services and staff.

**Conclusions:**

Most healthy childbearing women want a positive birth experience. Safety and psychosocial wellbeing are equally valued. Maternity care should be designed to fulfil or exceed womens’ personal and socio-cultural beliefs and expectations.

## Introduction

Optimum outcomes for pregnant women and their babies depend on acceptable, affordable, accessible, high quality provision of maternity care during pregnancy, childbirth, and the postnatal period [1]. However, the overuse of interventions in some contexts, and the underuse in others [2], along with growing evidence of disrespectful and abusive behaviors in some institutional settings [3,4] demonstrates that many maternity services are not meeting these standards. Good quality intrapartum care is vital, both for women and babies who are healthy, and for the minority who experience complications. Basing maternity service design and care provision on what women want and need is essential to maximize uptake of, and continuing access to, service provision [5]. If local maternity care provision is limited, women may report that they are satisfied, even if they have had poor quality care, as they will not be aware of any better alternatives. Finding out what matters to women about labour and birth (rather than only asking about their actual experiences of intrapartum care) offers the potential to establish what women value, irrespective of what is actually on offer. This could provide a basis for service improvement, locally, and internationally.

Transformational health care, as envisioned by the Global Strategy for Women’s, Children’s and Adolescent Health [6], requires maternity services to go beyond survival during childbirth. Understanding what outcomes are important to women is critical to developing clinical guidelines and policies that are women-centered, and that are more likely to ensure that women, babies and families thrive as well as survive following childbirth, with the ultimate aim of positive transformation of their lives, and those of their families and communities, in the short and longer term. The objective of this review was, therefore, to explore what matters to healthy women in relation to labour and birth. The findings have informed the framing and development of WHO intrapartum guideline recommendations, and the scope of outcomes to assess optimal intrapartum maternity care in future.

## Methods

We conducted a systematic qualitative review in accordance with the PRISMA guidelines (See S1 Table for PRISMA Checklist). We included studies where the focus was on healthy pregnant women, who are the majority of those accessing intrapartum care around the world. Study assessment included the use of a validated quality appraisal tool [7]. Meta-ethnographic techniques [8] were used for analysis and synthesis, and GRADE-CERQual [9] was applied to the resulting themes.

### Reflexive note

In keeping with quality standards for rigor in qualitative research [7] the review authors considered their views and opinions on intrapartum care as possible influences on the decisions made in the design and conduct of the study, and, in turn, on how the emerging results of the study influenced those views and opinions. All authors believed at the outset that most maternity care around the world is currently designed to maximize efficiency and to manage risk through precautionary interventions, with less emphasis on the experience of labour and birth for the mother, baby, and attending birth companions. All believed that positive labour experiences are important for the wellbeing of the mother, baby, and the family, in the short and longer term. Refutational analytic techniques [8] were therefore used to minimize the risk that these pre-suppositions would influence the analysis and the interpretation of the findings.

### Search strategy

**Fig 1:**
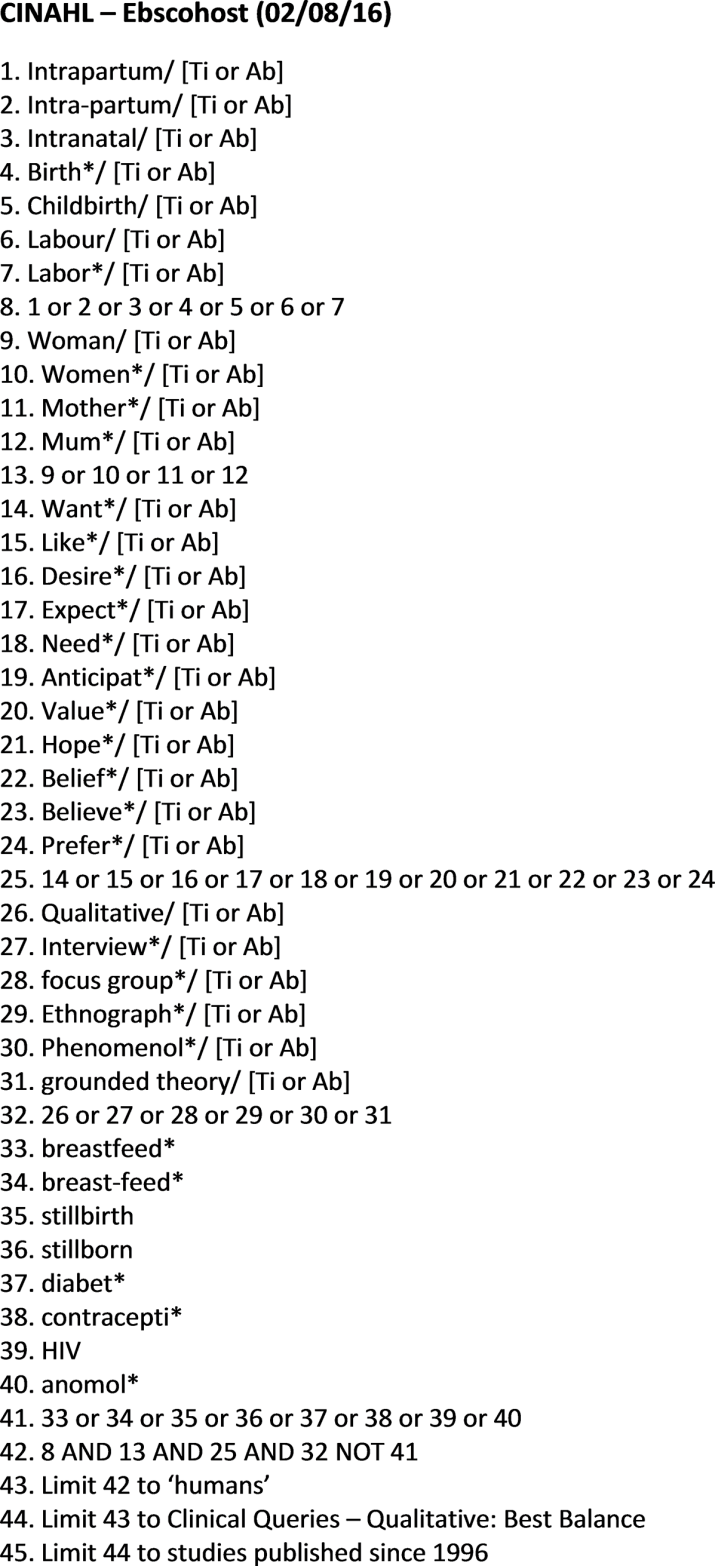
Search strategy [example].

An example of the search terms used is given in Fig 1.

In summary, the search terms were run in four broad strings covering population, intervention, outcome, and study type, with a view to capturing a wide selection of relevant studies. The terms were developed following a number of *a priori* scoping exercises across several databases. Where possible, relevant qualitative research limiters were used (e.g. Clinical Queries—Qualitative: Best Balance) to ensure that searches for qualitative studies were optimized. In instances where preliminary searches generated more than 3000 hits the Boolean operator ‘NOT’ was used to exclude studies that were unlikely to relate to the topic of interest. For example, NOT breastfeeding or breast-feeding or diabet* or contracepti* or HIV or anomol* [Ti,Ab]

### Inclusion/Exclusion criteria

No language restrictions were applied. Titles and/or abstracts of potentially relevant studies published in languages other than English were initially translated using a basic translation package (Google Translate). If this process suggested the study would be relevant, the full text was translated in detail by bi or multi-lingual colleagues at The University of Central Lancashire (UCLan) or the World Health Organization (WHO).

Studies published before 1996 were excluded, to ensure that the findings reflect the current generation of women who encounter modern intra-partum care. Only studies where the main focus was the beliefs and expectations of women about labour and childbirth (and not studies where the intent was to collect reflections on intrapartum services actually provided) were included. Studies were included if they reported on women’s views directly (and not through staff or partner opinion, or observational data), and where the views were of the general population of healthy women. Studies were excluded if they focused on a particular intervention (e.g epidural use) or procedure (e.g. episiotomy) or represented the views of specific subgroups of women with particular health problems (e.g. obesity, diabetes, pre-eclampsia, etc;). The views of women who were expecting to have a caesarean section for clinical reasons were also excluded.

KF screened the initial hits against the inclusion criteria and referred any queries to SD for discussion. Abstracts and full text papers were included based on consensus between KF and SD.

### Data sources

We searched the following databases: Medline, CINAHL, PsycINFO, AMED, EMBASE, LILACS (for studies conducted in South America) and AJOL (for studies conducted in Africa). Searches were conducted between 25^th^ July and 4th August 2016. Reference lists of included papers were scrutinized (backchained) and included as appropriate. Zetoc alerts were set up for over 50 relevant journals. Details of included papers were logged on a study specific excel file. An updated search was carried out for papers published between August 2016 and January 2018. The results were used as a confirmability check for the original findings.

### Quality assessment

The included studies were subject to quality appraisal using the instrument developed by Walsh and Downe [7] and modified by Downe et al [10]. This is a simple appraisal system that rates studies against 11 criteria, and then allocates a score from A-D to each study, based on the extent to which it demonstrated credibility, transferability, dependability, and conformability.

Studies scoring D (‘Significant flaws that are very likely to affect the credibility, transferability, dependability and/or confirmability of the study’) were excluded on quality grounds. [See S1 Appendix for details of Quality Assessment]

### Analytic strategy

The analytic process followed the method of Noblitt and Hare [8], which is derived from the constant comparison method [11]. In step one, the included papers were examined, and an index paper was selected, chosen to best reflect the focus of the review [12]. The themes and findings identified by the authors of this paper were entered onto a spreadsheet, to develop an initial thematic framework. The findings of all the remaining papers were then mapped to this framework, which continued to develop as the data from each paper were added [13]. This process includes looking for what is similar between papers (‘reciprocal analysis’), and for what contradicts (‘disconfirms’) the emerging findings (‘refutational analysis’). For the refutational process, as we added each included paper to the analysis, we consciously looked for data that could disconfirm our emerging themes, or our prior beliefs and views related to the topic of the review. If any disconfirming data were found, the themes were amended, so that they continued to capture all the data from the papers we had already analyzed, as well as taking account of the new insights. This process also ensured that the final analysis had high explanatory power for all the data. [See S1 Appenedix for details of thematic development]

The themes were all agreed by consensus between KF and SD, and subject to appraisal by all members of the review team. All were directly derived from quote material in more than one of the included studies. They were assessed for confidence in the quality, coherence, relevance and adequacy of the data contributing to them using the GRADE-CERQual tool [9]. This is a recently developed instrument, derived from the Grading of Recommendations, Assessment, Development and Evaluation (GRADE) approach used in quantitative effectiveness reviews. The GRADE-CERQual assessment results in a final classification of confidence in the theme in four categories: ‘high’, ‘moderate’, ‘low’ or ‘very low’. [See S1 Appendix for details of CERQual assessments]

All the themes were translated (or synthesized) into a ‘line of argument synthesis’ [8], based on theoretical concepts that explained the data at a conceptual level. The line of argument is more than the sum of the parts of the review. A robust line of argument has high theoretical transferability beyond the particular included studies, and so it is likely to be applicable in a wider range of settings and circumstances. The line of argument formed the basis for a Statement of Findings that was then used to inform the ‘values’ component of the Evidence to Decision frameworks used as the basis of the development of the WHO Intrapartum Care guideline (2018).

## Results

### Included studies

**Fig 2:**
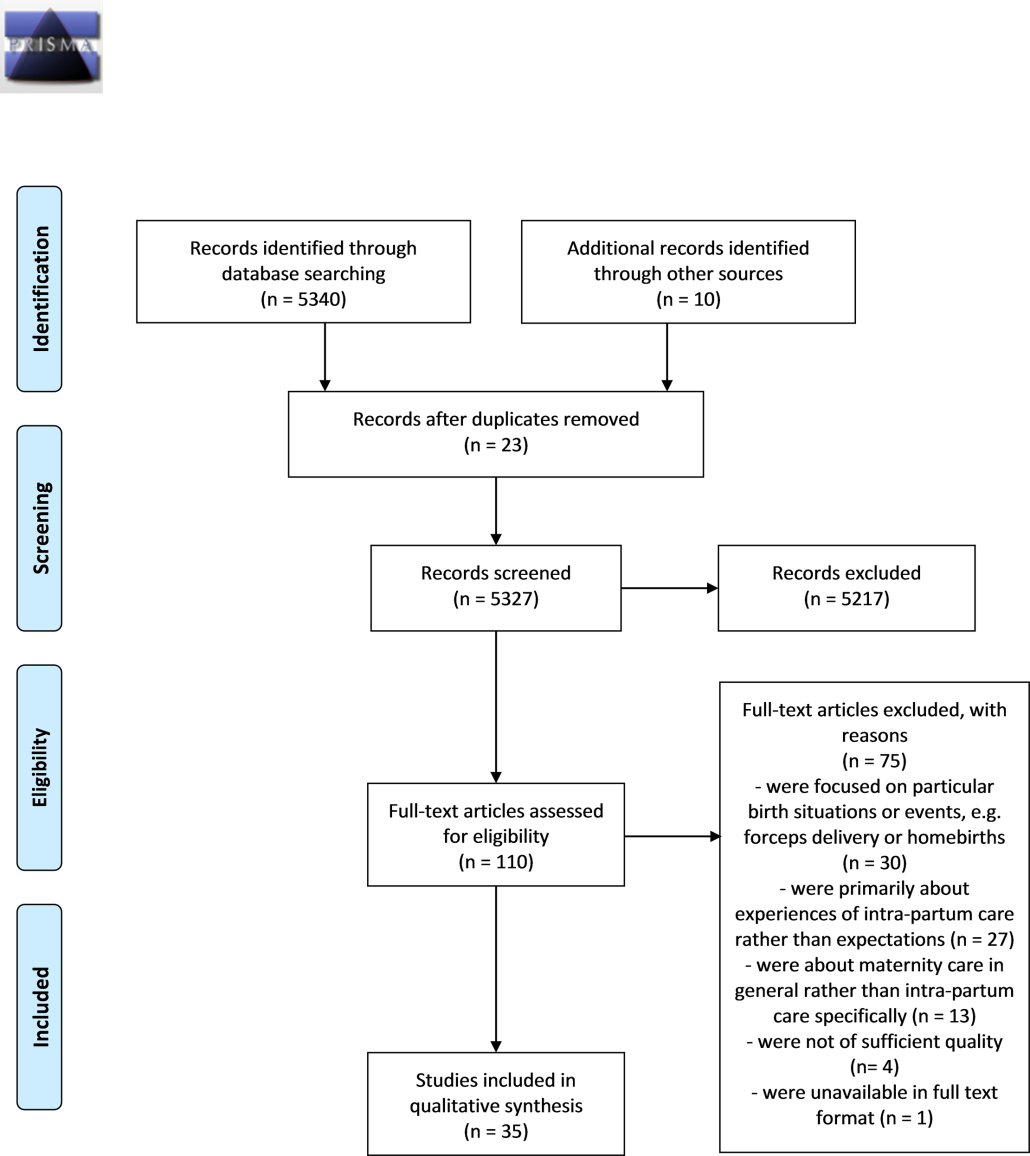
PRISMA flow diagram.

The primary search strategy generated a total of 5350 hits, including 10 already known to the authors. Twenty-three duplicate studies were removed, leaving 5327 to be screened. 5217 of these studies were excluded by title or abstract, primarily because they were deemed to be unrelated to the topic of interest. The remaining 110 were taken forward for full text review. A further 71 were excluded at this stage. The reasons for exclusion are shown in Fig 2.

Of the 39 full text papers, four were excluded after quality appraisal [14–17]. Two were relatively small Brazilian studies with little or no methodological information [14, 15], one was a mixed methods review with limited qualitative data [16] and one had limited methodological information[17]. Thirty five papers were included in the final analysis. Post-hoc examination of the four papers excluded on quality grounds indicated that inclusion of the data within them would not have changed the final themes, line of argument, or Summary of Findings statement.

There were no additional studies from the Zetoc alerts.

The updated search generated 26 hits (after screening by title) and a further 2 studies were identified [18, 19].

#### Characteristics and quality of included studies (primary search)

The characteristics and quality of the 35 included studies were tabulated, and are summarized in Table 1.

**Table 1:** Included studies: Characteristics and quality rating (primary search).

Code	Authors	Setting	Study design	Participant selection	Sample size	Date	Country	Quality
2	Callister, Holt & West Kuhre	Urban	In depth interviews	Convenience sampling	Interviews with 17 women	2010	**Australia**	B+
3	Fenwick et al	Urban/rural	Telephone interviews	Random based on response to a newspaper advert	202 women's narratives: open ended survey question	2005	**Australia**	A-
4	Maier	Urban	Feminist perspective, interviews	Unclear—randomly recruited at an antenatal clinic	Interviews with 27 women	2010	**Australia**	C+
5	Hauck et al	Urban/rural	In depth interviews	Purposive sampling with women from phase 1 of the study (see 15)	Interviews with 20 women (11 multiparous & 9 primiparous)	2007	**Australia**	B+
6	Malacrida & Boulton	Largely urban	Feminist Foucaultian ethnographic	Variety of methods including e-mail, adverts and snowball sampling	Interviews with 21 women	2014	**Canada**	B+
7	Malacrida & Boulton	Urban	Social constructionism, feminist, interviews	Brief details incorporating random (via e-mail and adverts) and snowball sampling	Interviews with 22 women	2012	**Canada**	B
8	Callister, Eads & Diel	Urban	In-depth interviews	Snowball sampling	34 women of Chinese origin	2011	**China & USA**	B
9	Murray	Urban	Ethnographic: observations, interviews, field notes	Unclear—researcher worked with informants over a 1 year period	Repeated interviews, 16 women, pre & post-natal	2012	**Chile**	C+
10	Melender	Urban and rural	In-depth interviews	Purposive sampling; rural and urban locations, low and high risk women	Interviews with 24 pregnant women	2006	**Finland**	A-
11	Halldorsdottir & Karlsdottir	Urban	Phenomenological interviews	Unclear—'through a network of colleagues'	Reflexive interviews with 14 women	1996	**Iceland**	B-
12	Aune et al	Urban	In depth interviews informed by salutogenic theory	Selective sampling; women who self- identified with positive birth experience	Interviews with 12 women who had given birth	2015	**Norway**	B+
13	Lundgren	Urban	Phenomenological in-depth interviews	Unclear—randomly selected group from a purposive sample via another study	11 women (5 primiparous & 5 multiparous)	2005	**Sweden**	B-
14	Rilby et al	Urban	Open ended survey	Follow up to a larger survey based study using prospective sampling	908 women (respondents to open ended survey)	2012	**Sweden**	B-
15	Brodrick	Urban	In-depth interviews	Random; those meeting inclusion criteria	8 women (all primiparous)	2008	**UK**	B
16	Gibbins & Thomson	Urban	Husserlian Phenomenology	Purposive	8 women, pre/ postnatal	2001	**UK**	A-
17	Proctor	Urban	Focus groups and interviews	Limited detail—maximum variation	33 women (19 pre and 14 post-natal) [and 47 staff]	1998	**UK**	C+
18	Highsmith	Urban	In-depth interviews based on pre-natal drawings of participants 'ideal birth'	No details	8 women interviewed before and after birth	2006	**USA**	B+
19	Martin, Bulmer & Pettker	Urban	Husserlian phenomenology, (semi-structured interviews)	Non probability purposive sampling at an antenatal clinic	Interviews with 7 women	2013	**USA**	B
21	Marin et al	Urban	In depth interviews	As part of a longitudinal study	Interviews with 7 women	2009	**Brazil **	C+
22	Pinheiro & Bittar	Urban	Interviews and observations	No details—random?	25 pregnant women and 2 postnatal mothers	2013	**Brazil **	C+
23	Dias & Deslandes	Urban	Interviews and ethnographic observations	Unclear—purposive sample of multiparous women at a prenatal clinic	Interviews with 22 women	2006	**Brazil **	C+
24	Nakano et al	Urban	Social constructionism; in-depth interviews	Convenience: women attending a vaccination clinic at 1 month post-natal	20 women, 1 month post-natal, attending a vaccination clinic	2012	**Brazil **	B
25	Raven et al	Rural	Interviews and focus groups	Purposive sampling at a variety of hospitals but no details	35 individual interviews, 5 focus groups (69 women)	2015	**China**	B-
26	Craig & Kabylbekova	Urban	Focus groups	Random, then snowball sampling	2 focus groups (21 women)	2015	**Kazakhstan**	B-
27	Chuahorm et al	Semi-urban	Grounded theory: interviews, field notes & observations	Purposive initially followed by theoretical sampling based on participant characteristics	20 women interviewed 48 hours after delivery and again 1 month later	2007	**Thailand**	B
28	Sercekus & Okumus	Urban	Interviews	Purposive sample of women attending an outpatient clinic	Interviews with 19 women	2009	**Turkey**	B-
29	Callister et al	Urban	Ethnographic principles: interviews and observations	Unclear—appears to be random on the post-natal ward	Interviews with 32 women	2010	**Ecuador**	B
30	D'ambruoso, Abbey & Hussein	Semi-urban	Qualitative, focus groups and latterly interviews	Opportunistic as part on a wider study	2 focus groups with women and a further 21 interviews	2005	**Ghana**	B
31	Wilkinson & Callister	Rural	Ethnography (health belief model): interviews, observations, field notes	Random selection at clinic then snowball sampling to women in outlying villages	Interviews with 24 women	2010	**Ghana**	A-
32	Corbett & Callister	Rural	Unclear—ethnographic?	Initially convenience in post-partum unit followed by snowball sampling	Interviews with 22 women	2012	**India**	B+
33	Sharma, Christenssen & Johansson	Rural	Grounded theory: focus groups, interviews field notes & observations	Unclear	Focus groups with 85 women (childless, pregnant and mothers)	2012	**India**	A-
34	Okwako & Symon	Urban	Phenomenological interviews	Convenience sample at an antenatal clinic	14 In depth interviews with 7 women (pre & post-natal)	2014	**Kenya**	B+
35	Gomi	Rural	Qualitative, some ethnographic approaches	Unclear	In depth interviews; 9 women: & stakeholders	2013	**Bangladesh**	C
36	Kaphle et al	Rural	Social constructionist incorporating theories of oppression and feminism	Purposive sample of a variety of participants in a remote location	Interviews with 25 pregnant or post-natal women and associated stakeholders	2013	**Nepal**	A-
37	Regmi & Madison	Rural	Unclear—In depth interviews	Unclear—described as purposive but no details of recruitment or strategy	Interviews with 15 women & 8 mothers-in-law; focus group (8 women);	2009	**Nepal**	C+

The date range of publication for the results of the primary search was 1996–2015. All regions of the world were represented. By continent, the largest number of studies were based in Europe (n = 9) [20–28]^,^ (UK x3, Sweden x2, Finland, Iceland, Norway, Turkey), and Asia (n = 9) [29–37], (China x2, India x2, Nepal x2, Bangladesh, Kazakhstan, Thailand). Six were from South America [38–43], (Brazil x4, Chile, Ecuador), four from North America [44–47], (Canada x2, USA x2), four from Australasia [48–51] and three from Africa [52–54], (Ghana x2, Kenya).

Most data were collected by individual interviews and/or focus or discussion groups. The papers incorporated a range of methodological approaches from relatively small phenomenological studies, to qualitative analysis of free text survey responses. They represented the views of more than 1800 women, from a wide range of ethnic backgrounds, ages (14–49) and socio-demographic groups. The quality was mostly moderate to high (B or above).

The eligible papers from the updated search were scrutinised to assess similarities or differences between the results generated from the primary review, and the themes and findings in the more recent studies.

### Findings

Table 2 presents the themes emerging from the synthesis of the data, along with codes, subthemes, and related quotes from the included studies, and the GRADE-CERQual rating of the sub-themes (‘evidence statements’). The numbers used in this table are indexed to the appropriate study in superscript in the reference list below.

**Table 2:** Themes emerging from the data (primary search).

Codes	Relevant Studies	CERQual Grading	Supporting data	Subthemes	Summary themes
**Belief in a 'Normal Birth' (without medical intervention).**	**18 Studies**– 20, 22, 23, 27, 30, 33, 37, 39, 41, 42, 44, 45, 47, 48, 49, 50, 51, 54	High	*It will be a challenging experience both physically and emotionally but yes*, *I guess my expectations are to be able to give birth naturally with the least amount of intervention*^*49*^	There is value in the experience of using one’s own physical and psychosocial capacities to labour, and to give birth to a healthy baby	***Hope for a positive birth experience; anticipating triumph and delight*, *fearing pain and abandonment***
**Want a healthy baby -**	**15 Studies**– 21, 24, 37, 39, 40, 42, 43, 45, 46, 47, 49, 50, 51, 52, 53	High	*…*.*after-all what is important is the goal of the activity*: *to come out as a live and healthy mother with a healthy baby*^*52*^.		
**Belief in the transcendence of birth**	**6 Studies**– 24, 43, 44, 46, 48, 54	Low	*I didn’t think [about childbirth being a spiritual experience] at the time*, *but looking back I do*. *I think it’s just the way you connect with your baby in a way that you’ve probably never connected with anything*. *It gives you a whole different perspective on your spiritual side*^*48*^		
**Want to hold their baby**	**2 Studies**– 45, 46	Very Low	*I didn’t get to hold my baby right away*, *I didn’t get to look into her eyes undrugged for who knows how long*. *I did get to hear her cry for the first time*, *but still*, *it was kind of delusional with all the medication and everything else … I started crying and thinking*, *‘I really*, *really missed out’*^*45*^		
**Fear of Childbirth**	**13 Studies**– 20, 21, 24, 31, 36, 37, 39, 40, 41, 46, 49, 51, 53	High	*I'm worried about delivery*. *This worries me every day*!.* *.* *.* *. *And they say 'but do not think about it*!*'*. *I say*: *I doubt there's a pregnant woman who does not think of the time of delivery*!^*39*^	Birth is inherently unpredictable, potentially frightening and usually painful: these emotions and sensations can be increased or diminished by expectations or experiences of care provision	
**Expect to go with the flow**	**7 Studies** –20, 23, 27, 44, 47, 49, 51	Moderate	*I know what my ideal is however I may need something else for the pain*, *it may not go to plan… I will just go with the flow*^*20*^		
**Expect labour and birth to be painful**	**7 Studies**– 21, 24, 28, 37, 46, 47, 48	Moderate	*I was afraid that my birth might be so difficult that I would need a caesarean birth*. *I was afraid of exhaustion* … *I was afraid of pain from being cut*. *I feared that it would be painful after birth*^*37*^		
**No expectations**	**2 Studies**– 21, 26	Very Low	*You see*, *it all came as a surprise to me*. *The birth itself and everything*. *But I presume it is only in the first birth*, *with the first baby you never know really what to expect*^*26*^		
**Value the support of a birth companion**	**14 Studies**– 20, 21, 22, 25, 26, 29, 30, 36, 38, 39, 41, 45, 46, 47	High	*What matters is that he’ll just be there* … *and as I actually said to my husband*, *now that I think of this childbirth*, *I want you to be there just as last time* … *that you hold me so that I can hide there* … *I mean* … *I wouldn’t have coped without him*^*25*^.		
**Want continuity of carer**	**4 Studies**– 21, 22, 30, 42	Low	*I think it would be good to be looked after in labour by a midwife I know*. *I like the midwives I have seen*. *There is one in particular I feel as if I have got on quite well with her*. *She is very reassuring and I think it would be ideal if she were going to be there*, *then that would be great*. *But she won't*, *it will be a different team of people*^*22*^		
**Need for a safe and supportive environment**	**12 Studies**– 20, 25, 26, 27, 28, 30, 32, 45, 46, 52, 53, 54	High	*At a certain stage [in the birth experience] you start to want*, *you start to yearn for this caring*, *you start to yearn for*. *somehow you are exhausted and you have been doing this for so long and then you just want someone to be kind to you and you know*., *feel sorry for you and just help you*. *You just realise that you can't do this alone and then you need a midwife*., *who you can feel is dedicated*., *that encourages you and* …*tells you that you can do it*, *that you are doing OK that it will be OK*.^*26*^		
**Fear of being alone**	**10 Studies**– 24, 27, 28, 32, 36, 37, 38, 39, 41, 54	Moderate	*Last time I was left alone and I did not have my sister*, *mother or anybody around*. *My husband was turned away*^*54*^		
**Expectations influenced by family and friends.**	**14 Studies**– 27, 28, 29, 30, 33, 34, 39, 41, 42, 44, 47, 49, 50, 53	Moderate	*My mum had five children…my mum told us about being born…*.*she had four home births and one hospital birth*, *so she told me what it was like and what she has gone through so I think that was a big influence*^*49*^	Familial and societal norms	***The enduring influence of familial and sociocultural norms and beliefs***
**Expectations influenced by media.**	**4 Studies**– 23, 46, 47, 49	Very Low	*I always go on the Internet*, *I signed up for all those pregnancy weekly kind of things*, *and I [sic] always constantly reading my book and everything*, *and to see about stuff that can happen*^*47*^		
**Acknowledgment of traditional beliefs.**	**5 Studies**– 29, 31, 33, 34, 53	Moderate	*if you insult people who are witches*, *they can plot against you during your* [pregnancy] *and they can fight at your birth*^*53*^	The power of cultural beliefs	
**Expect staff to be sensitive, caring and kind:**	**17 Studies**– 20, 22, 23, 24, 25, 26,30, 31, 37, 40, 41, 42, 47, 51, 52, 53, 54	High	…*She [Midwife] was great*, *she looked straight into my eyes and came to me and touched me warmly*, *in a personal way* …*like she was saying 'I am with you'* …*you know*, *an empowering touch which makes you stronger because you can sense that someone is with you in this* … *The birth progressed very fast after she came*, *incredibly faster than before* … *and I did not suffer such torment as before she came*^*26*^	Staff philosophies, attitudes, behaviours and skills are critically important	***Enacting what matters in the context of what is available***
****Fear of staff being distant, insensitive or rude.****	****8 Studies**–** 20, 24, 28, 37, 38, 42, 50, 52	Moderate	*It was inhumane*. *I came [to a public hospital] with high blood pressure (*…*) the obstetrician started with a vaginal exam and from then onwards I had contractions every five minutes (*…*) the older women in the room having their second child told me “Don’t listen and don’t say anything because these fools mistreat you when you complain*.*” I kept quiet and fainted a few times but no one noticed*. *I heard a midwife telling the woman next to me saying*: *“What do you complain about*? *Didn’t you like “it”*?^*42*^		
**Expect health professionals to be skilled and competent.**	**6 Studies**– 22, 24, 25, 30, 32, 51	Moderate	*They should be good at watching labour*, *doing the delivery*, *and abdominal delivery*. *Very good skills*. *The doctor must not hurry and can control everything*, *making me feel relaxed*^*30*^		
**Fear of medical intervention.**	**4 Studies**– 28, 32, 45, 53	Low	*I really*, *really was opposed to being induced–I feel that that is the beginning of the end for natural childbirth*^*45*^	Childbirth interventions or external controlling forces feared or welcomed	
**Want to have medical intervention(s).**	**6 Studies**– 28, 30, 41, 44, 50, 51	Low	*They are telling me to have a normal delivery*. *I don’t want a normal delivery*, *I’m really scared*, *I want a caesarean*, *have them put me to sleep and when I wake up I want my baby next to be*. *That’s what I want*.^*28*^		
**Hope for a quick labour.**	**6 Studies**– 21, 25, 29, 38, 45, 54	Low	*I mean that if childbirth would be prolonged for some reason*, *the child not beginning to arrive*… *as some have been in labor for 48 hours or so*, *I hope of course that it would be easier for me*^*25*^		
**Handing over control (to medical staff/God).**	**10 Studies**– 20, 23, 33, 38, 41, 42, 43, 45, 50, 53	Moderate	*You have to pray so that you leave everything to God*, *so that God will take control*… *During that time*, *everything is in prayer*. *You will be praying*… *every time*. *From the time that you knew that you were almost ready to deliver*, *you start praying and you make your mind set that you are going to give birth to the child*^*53*^		

The findings suggest that, with high or moderate confidence, most women around the world hope for a labour and birth experience that enables them to use their inherent physical and psychosocial capacities to labor and give birth to a healthy baby in a clinically, culturally, and psychologically safe environment with continuity of practical and emotional support from a birth companion(s), and with kind, sensitive clinical staff, who provide reassurance and technical competency. Most women place a high value on their capacity to give birth physiologically (expressed variously as ‘normal’ or ‘natural’, or without technical or pharmacological interventions) for the short and longer term physical and psychological wellbeing of themselves, their baby and their family; however, they also acknowledge that birth can be an unpredictable and potentially frightening event, and that they may need to ‘go with the flow’. Even where intervention is needed or wanted, women usually wish to retain a sense of personal achievement and control by being involved in decision making.

This is summarized in three overarching themes: *Hoping for a positive birth experience*: *anticipating triumph and delight*, *fearing pain and abandonment*; *the enduring influence of familial and socio- childbirth norms;* and *Enacting what matters in the context of what is available*.

These themes generated the following line of argument:

For most childbearing women across the world, there is inherent value in being able to use one’s own physical and psychosocial capacities to labour, and to give birth to a healthy baby, even when the process is unpredictable and painful. Beliefs about what matters to women are influenced by familial experiences, and local cultural norms and values. The capacity for women to enact what matters to them is affected by anticipated or actual encounters with maternity care staff and services, including the use of desired, required, and/ or feared childbirth interventions.

The themes and findings in the papers included in the updated search confirmed the review findings, suggesting that the analysis is robust, and theoretically transferable to a range of women and settings around the world.

## Discussion

For most of the respondents in the included studies, childbirth was an important experience, which had characteristics of what has been termed ‘liminality’: the transition stage between one state and another during a life-changing rite of passage [55]. For a small minority, childbirth was simply a physical process that should be conducted as quickly and painlessly as possible. As with other life-transition experiences, many women were fearful in anticipation of the hard work, pain, and uncertainty of labour, but most of them accepted these (potentially extreme) difficulties as part of the necessary process of achieving a positive, or even transformatory, birth experience for themselves and for their baby. Whatever they thought about the nature of birth, women interpreted their expectations of what could and should happen through the lens of family birth stories, and cultural and social norms. Whether women wanted birth over as quickly and painlessly as possible, or whether they understood it as fundamental to their transition to motherhood, they recognized the potential vulnerability of themselves and their baby through the process, and the essential uncertainty about what might happen. This was associated with a strong desire for safe, supportive, kind, respectful and responsive care during labor and birth. These characteristics applied to birth companions, professional and lay care givers, and to the processes and environment of care. The extent to which women could experience what mattered to them was mediated by the nature of the local maternity care provision that was available to them, including the attitudes and behaviours of staff, the quality of the relationship between women and care providers, and the resources and atmosphere of the local facility.

To our knowledge, this is the first meta-synthesis of what matters to women for labour and birth, as opposed to studies of women’s experiences once they have been through the process. Systematic reviews are inevitably dependent on the nature and quality of data that have already been collected and reported. In reviews of qualitative studies, these data have already been interpreted through the lens of what is seen to be important by the primary authors. Too few studies, from too narrow a cultural context, can limit the external transferability of the findings. Although the intent was to only include studies that reported on womens’ *a priori* views and expectations about what matters to them for labour and birth, independent of any intrapartum care they may have received, in some cases participants views were inevitably informed by their actual experiences.

However, the findings are strengthened by the inclusion of a large number of studies, covering every region of the world, and by the confirmatory analysis carried out as a result of the updated search.

The use of translation software at the inclusion stage of the review could theoretically have led to the exclusion of some relevant papers. In the event, 4 studies (from the primary search) that were included as a consequence of software translation were in languages other than English (3 in Portuguese and 1 in Japanese). The findings of all of these papers were translated by fluent speakers of the relevant language, and they were consistent with the papers written in English. The final analysis was consistent for women in all regions of the world. GRADE-CERQual assessments indicated that confidence in most of the findings was moderate or high, reflecting the quantity and quality of the included studies, and the wide range of settings, viewpoints, and study types included.

The findings largely reinforce the prior beliefs of the authors, which could suggest that different reviewers might have come to different conclusions. However, this risk was limited by the conscious search for disconfirming data to test the emerging codes, subthemes, and main themes.

The findings apply directly to healthy women of a range of parity, and in a range of cultural and economic settings, who are receiving routine intrapartum care. The review did not include studies that were only focused on women with specific health conditions, such as HIV or diabetes, or women from particular marginalised groups, such as those seen as ethnic or cultural outsiders, or very young or very poor women. However, women from some of these groups were part of the respondent sample in some of the included studies, and individual studies of the views of women who are marginalised suggest that the review findings are highly likely to be transferable [56–59].

Facility birth is generally accepted as a solution to persistently high rates of maternal and neonatal mortality and morbidity. However, since Bowser and Hill published their analysis of disrespect and abuse in institutional birth settings, in 2010 [60], there has been an increasing recognition that, while providing central facilities for maternity care is necessary for the provision of care to women and/or babies with complications, this strategy is not sufficient to ensure optimal outcomes for all women and babies [3]. Recent WHO antenatal guidelines incorporate evidence from qualitative systematic reviews, indicating that women value the psychological, cultural and emotional experience of pregnancy as well as the health of themselves and their growing baby [61, 62]. These reviews have also revealed that women experience pregnancy, birth, and the postnatal period as a psychological and physical continuum, and not as three distinct and un-related states. The current review adds to this body of evidence, by linking what women perceive as a positive labour and birth to local familial and cultural norms that shape the way that childbirth is framed, and by expressing the limitations on how far women believe they can actually enact a positive experience of labour and birth, depending on the available maternity care provision locally.

The findings support the multiple domains of the Lancet Quality of Maternal and Newborn Care Framework [5], and of the 2015 WHO Quality of Care Framework for Maternal and Newborn Health [1]. The former takes a human rights perspective, and incorporates a systematic review of what women want and need. The framework recognizes the importance of safe, accessible, evidence based, respectful care provision, and is based on a philosophy of care that optimizes physiological, psychological and cultural norms and values. The latter links the experience of care with provision of care, evidence based practices for routine care and management of complications, actionable information systems and functional referral systems, as well as competent and motivated human resources and essential physical resources.

The findings of this review also complement the Cochrane effectiveness reviews on midwife-led continuity of care [63] and continuous support in labour [64]. The finding that most women would prefer not to have labour interventions unless they are necessary for the safety of their baby and/or themselves is reinforced by the recent Lancet Maternal Health series, in which the excessive over-use of intrapartum interventions in both HIC and LMIC countries is shown to be potentially as serious a problem at the population level as the lack of availability of such interventions when they are life-saving [2].

## Conclusions

This review demonstrates that what matters to women in relation to childbirth is underpinned by three phenomena; the physical and psychosocial narture of birth as an embodied experience; local familial and socio-cultural norms that legitimate or reframe expectations about labour and birth; and how maternity care provision enables or restricts what matters. Whether women perceive childbirth to be a transformatory process that has meaning for them and their baby in the short and longer term, or whether they see it as a necessary process that should be completed as quickly and painlessly as possible, maternity services need to be responsive to their values, beliefs, and needs. What matters to women is also what is likely to generate the safest and most humanized maternity care provision, for mother, baby, and the family. There is now sufficient evidence from a wide range of sources to suggest that it is imperative that maternity services recognize the benefits of providing what matters to women (and the risks of not doing so). Crucially, these factors should become a central component of care provision as a matter of urgency to ensure the optimum uptake of effective and respectful maternity care, and, as a consequence, the health of childbearing women and their babies and families, in both the short and longer-term.

## Supporting information

S1 Appendix Quality assessment, data extraction and CERQual grading. (XLSX)

S1 Table PRISMA checklist. (DOC)
