# Correction: What matters to women during childbirth: A systematic qualitative review

**DOI:** 10.1371/journal.pone.0197791

**Published:** 2018-05-17

**Authors:** Soo Downe, Kenneth Finlayson, Olufemi T. Oladapo, Mercedes Bonet, A. Metin Gülmezoglu

The third author’s name appears incorrectly. The correct name is: Olufemi T. Oladapo. The correct citation is: Downe S, Finlayson K, Oladapo OT, Bonet M, Gülmezoglu AM (2018) What matters to women during childbirth: A systematic qualitative review. PLoS ONE 13(4): e0194906. https://doi.org/10.1371/journal.pone.0194906
